# Supplementary material for: Access to Health Information in the Polish Healthcare System—Survey Research
Source: Int J Environ Res Public Health. 2022 Jun 14;19(12):7320. doi: 10.3390/ijerph19127320 (PMC9223768; doi:10.3390/ijerph19127320)
Supplement: Supplementary file 1 [file ijerph-19-07320-s001.zip › ijerph-1739659-supplementary materials/Figure S1 Questionnaire.pdf]

* Required

1. How do you assess your health? Please check in the scale from 1 to 10, where 10 means the best health you can imagine, and 1 the worst health you can imagine. *

	1	2	3	4	5	6	7	8	9	10	
worst health	O	O	O	O	O	O	O	O	O	O	best health

2. Have you been diagnosed with a chronic illness / long-term ailment or disability? *

O yes

O no

3. If you have been diagnosed with a chronic illness, please specify for how long (if you have been diagnosed with more than one chronic illness / long-term ailment / disability, please check information on the one that was diagnosed first): *

O since birth / childhood

O for more than a dozen years, but the diagnosis has been given at an adult age

O for several years

O for a few months

O shorter than several months

4. What do you do after the occurrence of symptoms that may reveal an illness? You may check more than one answer *

☐ I consult a doctor

☐ I look for information at the chemist's

☐ I search for information over the Internet

☐ I ask my family

☐ I go to a witch doctor / healer

☐ I take no actions

☐ Other

5. How often do you search for information relating to health? *

O never

O hardly ever

O sometimes

O often

O very often

6. For what reason do you look for health information (you may check more than one answer): *

☐ prophylactically, when I want to prevent a disease

☐ I am interested in health issues

☐ due to symptoms of an illness that occur in me / my family member

☐ to verify a diagnosis given by a doctor

☐ because I have not received an answer to my question when I saw a doctor

☐ because I did not understand information given by a doctor

☐ other

7. What type of information do you look for (you may check more than one answer): *

☐ about symptoms of an illness

☐ about prescription drugs prescribed by my doctor

☐ about OTC drugs

☐ about vaccinations

☐ about therapeutic methods

☐ about surgical procedures

☐ about healthy lifestyle

☐ about dieting

☐ about using food supplements

☐ about issues relating to blood donation / transplantology

☐ other

8. Where do you obtain health information from?

If you use a given source, please check on the scale your level of satisfaction with information received. If you do not use a source of information, check the 'I do not use it' option. *

	I do not use it	Definitely unsatisfactory	Rather poor	I have no opinion	Rather good	Definitely good
from a doctor	O	O	O	O	O	O
at the chemist's	O	O	O	O	O	O

from a nurse	O	O	O	O	O	O
from the Internet	O	O	O	O	O	O
from advertisements	O	O	O	O	O	O
from television / radio programmes	O	O	O	O	O	O
from family members/ friends	O	O	O	O	O	O

9. If you get your health information from sources other than listed above, please specify sources:

10. If you look for health information over the Internet, pleas specify what sources you use <u>most often</u> (you may check more than one response)? *

☐ I never look for health information over the Internet

☐ Internet forums

☐ social media

☐ medical portals

☐ medical magazines available online

☐ blogs/vlogs of doctors

☐ I do not know, I choose sources suggested by a search engine

☐ other

11. Where would you like to get health information from if you were certain that such information is reliable and easily accessible (you may check more than one answer)? *

☐ from a doctor

☐ at the chemist’s

☐ from nurses

☐ on online forums

☐ in social media

☐ on medical portals

☐ in online medical magazines

☐ on doctor’s blogs / vlogs

☐ on television

☐ in radio

☐ from family members

☐ other

12. Do you ever fail to consult symptoms that may show an illness with a doctor? If yes, check the reasons (you may check more than one answer): *

☐ I <u>always</u> consult symptoms that may reveal an illness with a doctor

☐ I do not get consultation if ailments are mild

☐ I do not consult a doctor if ailments that occur are known to me and I know how to treat them myself

☐ I do not consult a doctor because of long waiting times and problems in access to a doctor

☐ I do not consult a doctor because I feel ashamed or I am afraid

☐ I do not consult a doctor because I want to save time and/or money

☐ I do not consult a doctor because of a long distance to a healthcare centre

☐ I neve consult a doctor, because I do not believe in modern medicine

13. Do you ever use a drug without consulting a doctor? *

O never

O hardly ever

O sometimes

O often

O usually

14. Do you ever modify the application of a drug or a therapeutic method suggested by a doctor under the influence of information received from other sources? *

O never

O hardly ever

O sometimes

O often

O usually

15. Do you use food supplements or other products available at the chemist's that are not drugs, without consulting a physician? *

O never

O hardly ever

O sometimes

O often

O usually

Demographic data

16. age *

O 18-25

O 26-40

O 41-60

O 61-80

O >80

17. Sex *

O woman

O man

O other / I do not want to answer this question

18. Education *

O primary

O junior secondary

O basic vocational

O secondary

O higher

19. Professional status *

O student

O unemployed

O employed

O old age pensioner

O pensioner

20. Place of domicile *

O village

O town with population of up to 50 thousand

O city with population of up to 100 thousand

O city with population of up to 250 thousand

O city with population of more than 250 thousand

21. Marital status *

O single

O in a permanent relationship

O married

O divorced

O widow(er)

22. How do you assess your economic situation? *

O very good

O good

O average

O poor

O very bad
