# Supplementary material for: Access to Health Information in the Polish Healthcare System—Survey Research
Source: Int J Environ Res Public Health. 2022 Jun 14;19(12):7320. doi: 10.3390/ijerph19127320 (PMC9223768; doi:10.3390/ijerph19127320)
Supplement: Supplementary file 1 [file ijerph-19-07320-s001.zip › ijerph-1739659-supplementary materials/Figure S2. Demographic characteristicsí¬Online respondents.pdf]

DEMOGRAPHIC DATA- ONLINE RESPONDENTS

Age

18-25	32
26-40	135
41-60	127
61-80	21

Sex

woman	202
man	113

Education

primary	2
junior secondary	1
basic vocational	3
secondary	36
higher	273

Professional status

old age pensioner	14
employed	270
pensioner	13
student	18

Place of domicile

town with population of up to 50 thousand	35
city with population of up to 100 thousand	14
city with population of up to 250 thousand	38
city with population of more than 250 thousand	200
village	28

Marital status

divorced	23
single	66
in a permanent relationship	61
married	161
widow(er)	4

How do you assess your economic situation?

very good	70
good	157
average	85
poor	2
very bad	1
