# Supplementary material for: Access to Health Information in the Polish Healthcare System—Survey Research
Source: Int J Environ Res Public Health. 2022 Jun 14;19(12):7320. doi: 10.3390/ijerph19127320 (PMC9223768; doi:10.3390/ijerph19127320)
Supplement: Supplementary file 1 [file ijerph-19-07320-s001.zip › ijerph-1739659-supplementary materials/Figure S3. Demographic characteristicsí¬Offline respondents.pdf]

DEMOGRAPHIC DATA- OFFLINE RESPONDENTS

Age

18-25	3
26-40	7
41-60	14
61-80	17
>80	2

Sex

woman	28
man	14

Education

primary	6
junior secondary	2
basic vocational	16
secondary	11
higher	8

Professional status

old age pensioner	15
employed	18
pensioner	6
student	4

Place of domicile

town with population of up to 50 thousand	1
village	40

Marital status

divorced	3
single	5
in a permanent relationship	5
married	20
widow(er)	9

How do you assess your economic situation?

very good	2
good	8
average	20
poor	11
very bad	2
